# Metabolic score tool for personalized acute pancreatitis prognosis: A multicenter analysis

**DOI:** 10.17305/bb.2024.10222

**Published:** 2024-08-01

**Authors:** Shi-Jun Chen, Shu-Ling Wang, Chun-Sen Chen, Ying Xie, Yan-Ya Lin, Cun-Rong Chen, Jian-Xiong Hu

**Affiliations:** 1 Department of Critical Care Medicine, Affiliated Hospital of Putian University, Putian, China; 2 Department of Critical Care Medicine, Fujian Medical University Union Hospital, Fuzhou, China; 3 Department of Radiology, Fujian Medical University Union Hospital, Fuzhou, China; 4 School of Mechanical, Electrical and Information Engineering, Putian University, Putian, China; 5 The School of Clinical Medicine, Fujian Medical University, Fuzhou, China

**Keywords:** Acute pancreatitis (AP), body composition, prediction, severity

## Abstract

Increasing evidence suggests that body composition is associated with the development of acute pancreatitis (AP). This study aimed to investigate the applicability of body composition in predicting AP severity. Data of 213 patients with AP from the Affiliated Hospital of Putian University (AHOPTU) were included in this study, whilst data of 173 patients with AP from Fujian Medical University Union Hospital (FMUUH) were used for external validation. Patients were classified into the non-severe and severe groups according to AP severity. After seven days of treatment, in patients from AHOPTU, the difference in skeletal muscle index before and after treatment (ΔSMI) was significantly higher (*P* ═ 0.002), while the skeletal muscle radiodensity before treatment (PreSMR) was significantly lower (*P* ═ 0.042) in the non-severe group than in the severe group. The multivariate logistic regression model also revealed that the ΔSMI and PreSMR were independent risk factors for AP severity. The optimal cut-off values of ΔSMI and PreSMR were 1.0 and 43.7, respectively. The following metabolic score (SMS) was established to predict AP severity: 0: ΔSMI < 1.0 and PreSMR < 43.7; 1: ΔSMI ≥ 1.0 and PreSMR < 43.7 or ΔSMI < 1.0 and PreSMR ≥ 43.7; 3: ΔSMI ≥ 1.0 and PreSMR ≥ 43.7. In patients from AHOPTU and FMUUH, the areas under the curves for this model were 0.764 and 0.741, respectively. ΔSMI and PreSMR can accurately predict AP severity. It is recommended to routinely evaluate the statuses of patients with AP using the predictive model presented in this study for individualized treatment.

## Introduction

Acute pancreatitis (AP) is a digestive and injury-causing disease of the pancreatic tissues that has various etiologies. It is a common acute abdominal condition in intensive care medicine characterized by its rapid onset, swift progression, and severe impact. Currently, the global incidence of AP is approximately 13–45 cases per 100,000 individuals, and this rate is increasing worldwide. Reports indicate that in 20% of cases, AP progresses to severe AP (SAP), with multiple organ dysfunction, a grim prognosis, and a mortality rate of 13%–35% [1–3]. For AP, with its uncertain prognosis, it is important to avoid overzealous, premature, insufficient, or delayed interventions because inappropriate treatment may result in high recurrence rates, repeated exacerbation of the condition, and residual pancreatic secretory disorders, among other complications [4]. Therefore, it is crucial to dynamically assess the patient’s condition at the appropriate time and adopt a progressive intervention approach. Developing risk stratification tools to predict the AP severity that meets clinical needs is vital for guiding clinicians in resource allocation, patient counseling, and clinical auditing. This, along with multidisciplinary approaches that include evidence-based treatment, is vital to achieving optimal clinical outcomes [5].

Various prognostic scoring systems that combine clinical indicators have been developed; however, none possesses sufficient predictive power to enable clinicians to accurately assess the disease course and AP severity [6]. Similarly, the accessibility of information and the necessary variables for calculating scores also impact the timeliness of care provided to patients, which is critical in the context of resource allocation and nursing priorities [7]. Therefore, relatively faster and more accurate predictors have been widely investigated. In 2012, the Acute Pancreatitis Classification Working Group modified the Atlanta Classification System to enhance the AP assessment and treatment. This revised classification focuses on the morphological appearance of AP, making radiology increasingly important in the assessment and follow-up of patients with AP [8, 9]. Computed tomography (CT) has become the primary tool for AP monitoring and assessment, as well as the most widely used method for body composition assessment. It can describe various parameters associated with sarcopenic obesity, such as subcutaneous adipose tissue (SAT), visceral adipose tissue (VAT), skeletal muscle index (SMI), and skeletal muscle radiodensity (SMR). Previous studies have shown that sarcopenia is associated with a poor prognosis, complications, and an increased incidence of pancreatic and other cancers [10]. Furthermore, an increase in skeletal muscle mass has been shown to improve the resection rate of digestive tract tumors [11]. However, the impact of body composition-related parameters on AP outcomes has not been fully explored. Therefore, this multicentre study evaluated the applicability of body composition parameters, such as fat and muscle, in predicting AP severity.

## Materials and methods

### Study population and data collection

This study retrospectively analyzed data of 213 patients with AP who were treated at the Affiliated Hospital of Putian University (AHOPTU) between January 2010 and December 2021. The inclusion criteria were the following: (1) patients duly diagnosed with AP who were admitted within 24 h of onset, underwent emergency plain CT immediately upon admission, and underwent another plain CT on the seventh day after hospital admission for treatment and (2) patients in whom the etiology of AP was only biliary, hypertriglyceridemia-, alcoholic-, or drug-induced, or idiopathic. The exclusion criteria were as follows: (1) age <18 or >85 years; (2) diagnosis of pancreatic cancer or chronic pancreatitis; (3) pregnancy; (4) recurrent AP; (5) severe liver, kidney, respiratory, cardiovascular diseases, or malignant tumors; (6) local or systemic infection before the onset; and (7) incomplete important data or missing target variables.

A total of 173 patients with AP who sought care at the Fujian Medical University Union Hospital (FMUUH) from April 2014 to December 2021 were enrolled as an external validation dataset based on the same selection criteria.

**Figure 1.**
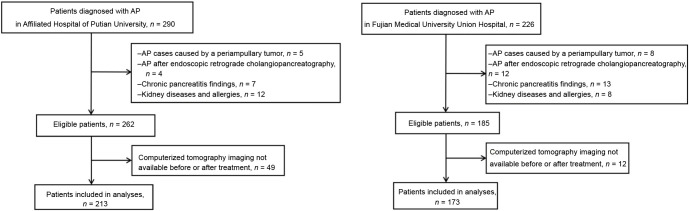
**Flowchart of the patient selection process.** AP: Acute pancreatitis; CT: Computerized tomography.

The screening process used in this study is illustrated in Figure 1. Clinical data was retrospectively collected from the hospitals’ healthcare systems.

### Definitions

Systemic inflammatory response syndrome (SIRS) was defined as the presence of at least two of the following factors: body temperature >38 ^∘^C or <36 ^∘^C; heart rate of >90 beats/min; shortness of breath, hyperventilation of >20 cycles/min, or PaCO_2_ of <32 mmHg; and white blood cell count of >12 × 10^9^/L or <4 × 10^9^/L. Organ dysfunction was defined using the modified Marshall scoring system used for the revised Atlanta classification. Acute respiratory failure was defined as an oxygen uptake concentration (FiO_2_) of 25% or the need for mechanical ventilation, but still with a PaO_2_ of ≤60 mmHg. Acute kidney injury was defined as a serum creatinine level of >1.9 mg/dL after fluid replacement or the need for renal replacement therapy (hemofiltration or hemodialysis). Circulatory failure was defined as a systolic blood pressure of <90 mmHg, unresponsiveness to fluid resuscitation, or systolic booster support. The clinical types of AP included mild AP (MAP), characterized by the absence of organ failure or local or systemic complications; moderate SAP (MSAP), characterized by transient organ failure (<48 h) or local or systemic complications; and SAP, characterized by persistent sexual organ failure (>48 h). Local complications of the condition were conditions such as pancreatic necrosis, pancreatic pseudocyst, and pancreatic abscess. Systemic complications included organ failure, bacteremia, acute respiratory distress syndrome, and shock.

### Nutritional support

The nutritional needs of the patients were managed by dietitians, and nutritional follow-up was conducted regularly in the ICU. Adhering to the principle of individualized treatment, the patients were administered oral nutrition in case of tolerance, whilst the intolerance resulted in nasogastric or nasoenteric feeding. For patients who were intolerant to enteral feeding, total parenteral nutrition was the alternative option. Energy requirements for both enteral and parenteral feeding were estimated using indirect calorimetry or calculated at 25–35 kcal/kg/day based on the following formula: protein 1.2–1.5 g/kg/day, carbohydrates 3–6 g/kg/day (corresponding to a target blood glucose concentration of <10 mmol/L), lipids up to 2 g/kg/day (corresponding to a target blood triglyceride concentration of <12 mmol/L), sodium 1–2 mmol/kg/day, potassium 1–2 mmol/kg/day, chloride 2–4 mmol/kg/day, and calcium 0.1 mmol/kg/day. Adjustments were made based on serum concentrations, metabolic statuses, and balance conditions [12].

### Body composition

**Figure S1.**
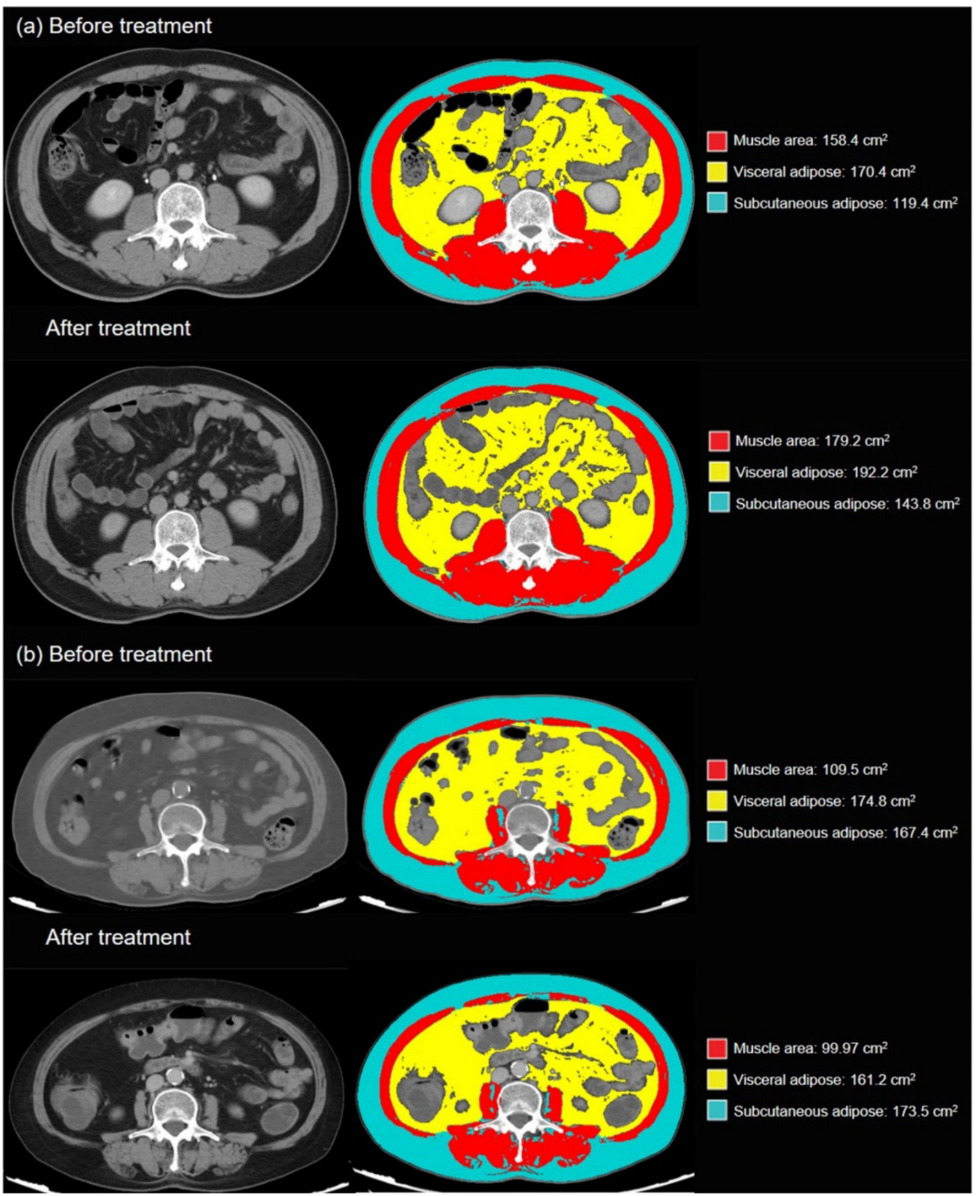
Body composition legend of (A) a 55-year-old male patient with non-SAP whose ΔSMI value is 6.8, (B) a 68-year-old female patient with SAP, whose ΔSMI value is −9.5. SAP: Severe acute pancreatitis; SMI: Skeletal muscle index.

Body composition information were obtained by taking CT images at the level of L3 vertebral since they can adequately reflect the distribution of fat and muscles in the whole body [13, 14]. Per the standard, the CT values of subcutaneous and visceral fat are −190 to −30 and −150 to −50 HU, respectively, while that of muscle is −30 to 150 HU. Two researchers (Shi-Jun Chen and Ying Xie) underwent prior intensive training, including training on accurate CT images at the L3 vertebral level acquisition as well as on accurate segmentation and calculation of different body compositions. The two researchers then used SliceOmatic software (version 5.0; TomoVision) to independently analyze CT data from 213 patients in the experimental group and 176 patients in the validation group. To avoid any bias in the analysis, they were blinded to any patient information. The researchers were required to measure the cross-sectional skeletal muscle area (cm^2^) at the L3 vertebral level. Two consecutive transverse sections that were both visible to the spine were used, and the average surface area (cm^2^) of the two consecutive sections was analyzed, normalized by height (m^2^), to obtain corresponding body composition information, such as the SAT, VAT, and SMI (cm^2^/m^2^) (Figure S1) [15].

### Ethical statement

The study was performed in strict accordance with the Declaration of Helsinki. The study was approved by the ethics committees of the Affiliated Hospital of Putian University (approval number: 2022068-XZ01) and FMUUH (approval number: 2023KY067). Informed consent was waived because of the retrospective nature of the study.

### Statistical analysis

Continuous variables are expressed as the mean ± standard deviation, whilst categorical variables are expressed as frequencies and percentages. The chi-square test, Fisher’s exact probability test, or the unpaired *t*-test were employed to compare clinicopathological data between the two groups, and a paired *t*-test was employed to compare clinicopathological data before and after treatment. All analyses were performed using SPSS version 23.0 (IBM Corp., Armonk, NY, USA) and RStudio version 1.1.419 (RStudio Inc.). Two-sided *P* < 0.05 was considered statistically significant.

## Results

### Demographics and baseline data

This study included 213 patients with AP who received treatment at the AHOPTU between January 2010 and December 2021 (Table 1). Among them, 109 (51.2%) were male and 104 (48.8%) were female. The mean age of the patients was 60 ± 10.3 years. Among the enrolled patients, 127 (59.6%) had a body mass index (BMI) of >25 kg/m^2^. There were 66 (31.0%) and 72 (33.8%) patients with diabetes and hypertension, respectively. Furthermore, 59 (27.7%) patients developed SIRS within 48 h of admission, 55 (25.8%) patients developed organ failure within 48 h of admission, 81 (38.0%) patients required ICU treatment, and 18 (8.5%) patients died during hospitalization. Table 1 summarizes the baseline characteristics of patients with FMUUH.

**Table 1:** Demographic and pathological characteristics of patients from AHOPTU and FMUUH

**Variables**	**AHOPTU (*n* ═ 213)**	**FMUUH (*n* ═ 173)**	***P* value**
Age, mean (SD), years	60 (10.3)	56 (8.5)	0.011
Sex, *n* (%)			0.100
Male	109 (51.2%)	103 (59.5%)	
Female	104 (48.8%)	70 (40.5%)	
ASA score, *n* (%)			0.508
≤2	141 (66.2%)	120 (69.4%)	
>2	72 (33.8%)	53 (30.6%)	
Body mass index, *n* (%)			0.085
≤25 kg/m^2^	86 (40.4%)	85 (49.1%)	
>25 kg/m^2^	127 (59.6%)	88 (50.9%)	
Diabetes, *n* (%)	66 (31.0%)	60 (34.7%)	0.441
Hypertension, *n* (%)	72 (33.8%)	68 (39.3%)	0.263
Alcohol, *n* (%)	104 (48.8%)	70 (40.5%)	0.101
Smoking, *n* (%)	77 (36.2%)	65 (37.6%)	0.773
WBC, mean (SD), ×10^9^/L	13.22 (5.31)	13.66 (5.78)	0.437
HCT, mean (SD), %	40.7 (5.5)	41.2 (5.6)	0.340
CRP, mean (SD), mg/L	77.39 (49.23)	78.34 (47.98)	0.791
PCT, mean (SD), ng/mL	4.52 (3.71)	4.67 (3.66)	0.887
Blood amylase, mean (SD), U/L	295.3 (132.8)	297.1 (129.8)	0.894
Blood lipase, mean (SD), U/L	367.2 (88.2)	369.1 (86.8)	0.832
Blood calcium, mean (SD), mmol/L	2.02 (0.21)	2.01 (0.30)	0.847
Etiology, *n* (%)			0.131
Alcohol	67 (31.5%)	54 (31.2%)	
Biliary	65 (30.5%)	68 (39.3%)	
Hypertriglyceridemia	14 (6.6%)	4 (2.3%)	
Drug-induced	9 (4.2%)	4 (2.3%)	
Idiopathic	58 (27.2%)	43 (24.9%)	
*Major complication, n (%)*			
ARDS	37 (17.4%)	32 (18.5%)	0.774
Bacteremia	36 (16.9%)	26 (15.0%)	0.618
Shock	27 (12.7%)	26 (15.0%)	0.504
Single organ failure	33 (15.5%)	31 (17.9%)	0.524
Multiple organ failure	23 (10.8%)	20 (11.6%)	0.813
Pancreatic necrosis	42 (19.7%)	40 (23.1%)	0.416
Pancreatic pseudocyst	45 (21.1%)	31 (17.9%)	0.431
Pancreatic abscess	15 (7.0%)	11 (6.4%)	0.790
Pancreatic endocrine or exocrine insufficiency	23 (10.8%)	20 (11.6%)	0.813
*Severity outcome*			
Persistent SIRS after 48h, *n* (%)	59 (27.7%)	51 (29.5%)	0.700
Persistent organ failure after 48h, *n* (%)	55 (25.8%)	41 (23.7%)	0.631
Need for ICU admission, *n* (%)	81 (38.0%)	50 (28.9%)	0.060
Length of stay, median [IQR], days	14 [5–29]	14 [5–26]	0.829
In-hospital mortality, *n* (%)	18 (8.5%)	9 (5.2%)	0.213
30 days unplanned readmission, *n* (%)	41 (19.2%)	28 (16.2%)	0.435
*Interventions performed, n (%)*			
Percutaneous puncture drainage	55 (25.8%)	52 (30.1%)	0.355
Endoscopic transluminal drainage/necrosectomy	12 (5.6%)	11 (6.4%)	0.765
Open necrosectomy	6 (2.8%)	4 (2.3%)	0.756

ASA: American Society of Anesthesiologists; WBC: White blood cells; HCT: Hematocrit; CRP: C-reactive protein; PCT: Procalcitonin; ARDS: Acute respiratory distress syndrome; AHOPTU: Affiliated Hospital of Putian University; FMUUH: Fujian Medical University Union Hospital; SIRS: Systemic inflammatory response syndrome.

Compared with patients from AHOPTU, those from FMUUH were relatively younger (56 years vs. 60 years), and the proportions of females and patients with a BMI of >25 kg/m^2^ were relatively lower in FMUUH (40.5% vs 48.8%, 50.9% vs 59.6%, respectively). Among patients from FMUUH, 51 (29.5%) of them developed SIRS within 48 h of admission, 41 (23.7%) developed organ failure, and 50 (28.9%) patients were admitted to the ICU. Nine patients (5.2%) died during hospitalization. There were no significant differences in risk factors of AP, such as smoking and diabetes, and major complications between the patients from AHOPTU and FMUUH.

### Characteristics of body composition during treatment

Table S1 shows the changes in body composition before and after treatment. Before treatment, the median BMI of patients from AHOPTU was 25.9 kg/m^2^ (IQR [23.5–28.2]), the median SAT was 29.5 cm^2^/m^2^ (IQR [15.1–44.7]), the median VAT was 21.3 cm^2^/m^2^ (IQR [5.9–41.2]), the median SMI was 47.2 cm^2^/m^2^ (IQR [40.7–51.9]), and the median SMR was 44.0 HU (IQR [40.3–49.5]). At CT review seven days after admission, the median BMI of the study population was 25.5 kg/m^2^ (IQR [23.5–27.9]), the median SAT was 28.2 cm^2^/m^2^ (IQR [18.3–43.2]), the median VAT was 20.3 cm^2^/m^2^ (IQR [9.9–39.1]), the median SMI was 46.7/m^2^ (IQR [41.2–53.2]), and the median SMR was 44.6 HU (IQR [39.7–48.7]). In general, there were no statistically significant changes in body composition during the seven days of treatment as all *P-*values were > 0.05 and Pearson correlation coefficients were > 0.6.

### Factors influencing AP severity

Patients with MAP or MSAP were included in the non-severe group, whereas patients with SAP were included in the severe group. Table 2 compares the levels of various body components in patients with AP based on different degrees of severity. The results showed that ΔSMI in the non-severe group was significantly higher than that in the severe group (1.0 vs −3.3, *P* ═ 0.002), and the non-severe group had a lower PreSMR than the severe group before treatment (42.5 vs 44.6, *P* ═ 0.041). There were no statistically significant differences in BMI, SAT, and VAT before and after treatment between both groups (*P* > 0.05). Variations in the muscle index before treatment (PreSMI), SMI after treatment (PostSMI), SMR after treatment (PostSMR), and the difference between SMR before and after treatment (ΔSMR) did not differ significantly (*P* > 0.05). Therefore, in this study, ΔSMI and PreSMR were included in the logistic regression model to analyze the factors that influence AP severity. Univariate comparisons showed that the white blood cell count, ΔSMI, and PreSMR significantly affected AP severity (all *P*-values were < 0.05). These factors were further included in the multivariate regression analysis, which revealed that ΔSMI and PreSMR were independent predictors of AP severity (Table S2).

**Table 2:** Relationship between changes in body composition before and after treatment among severity groups

**Variables**	**Non-severe group (*n* ═ 124)**	**Severe group (*n* ═ 89)**	***P* value**
*Body mass index, mean (SD), kg/m^2^*			
Before treatment	26.5 (3.2)	25.6 (2.0)	0.346
After treatment	26.0 (3.2)	25.6 (2.3)	0.866
Difference	−0.6 (2.4)	0.0 (1.3)	0.238
*Subcutaneous adipose tissue, mean (SD), cm^2^/m^2^*			
Before treatment	29.1 (18.3)	30.2 (15.7)	0.708
After treatment	28.0 (19.6)	28.4 (16.9)	0.867
Difference	−1.1 (8.7)	−1.8 (7.4)	0.648
*Visceral adipose tissue, mean (SD), cm^2^/m^2^*			
Before treatment	22.0 (23.5)	20.2 (19.6)	0.298
After treatment	21.8 (19.8)	19.5 (20.5)	0.803
Difference	−0.2 (10.8)	−0.7 (10.1)	0.084
*Skeletal muscle index, mean (SD), cm^2^/m^2^*			
Before treatment	46.0 (8.5)	49.5 (8.6)	0.690
After treatment	47.0 (8.9)	46.2 (8.1)	0.062
Difference	1.0 (4.0)	−3.3 (2.9)	0.002
*Skeletal muscle radiodensity, mean (SD), HU*			
Before treatment	42.5 (7.4)	44.6 (6.0)	0.041
After treatment	44.1 (7.0)	45.9 (7.3)	0.763
Difference	1.6 (5.7)	1.3 (6.8)	0.136

### Predictive value of the muscle index for AP severity

**Figure 2.**
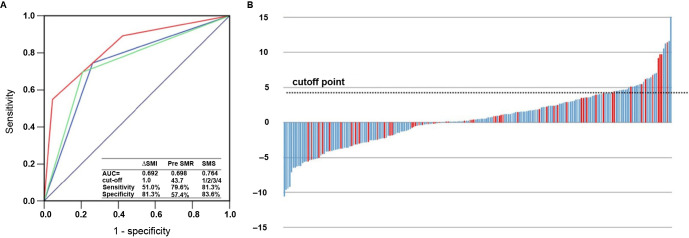
(A) ROC of a model predicting AP severity. Green line: ΔSMI ≥ 1.0, AUC ═ 0.692, blue line: PreSMR ≥ 43.7, AUC ═ 0.698, red line: SMS, AUC ═ 0.764. (B) Waterfall diagram showing ΔSMI in all patients. AP: Acute pancreatitis; SMI: Skeletal muscle index; ROC: Receiver operating characteristic; AUC: Area under the curve; SMS: Stroke mobility score; PreSMR: Skeletal muscle radiodensity before treatment.

The AUCs for ΔSMI and PreSMR were 0.692 and 0.698, respectively (Figure 2). Based on these results, we established a muscle score (SMS) to predict AP severity: 0: ΔSMI < 1.0 and PreSMR < 43.7; 1 score: ΔSMI ≥ 1.0, PreSMR < 43.7 or ΔSMI < 1.0, and PreSMR ≥ 43.7; 3 scores: ΔSMI ≥ 1.0 and PreSMR ≥ 43.7. In the AHOPTU population, the AUC for this indicator was 0.764.

### External validation of the model’s predictability

**Figure 3.**
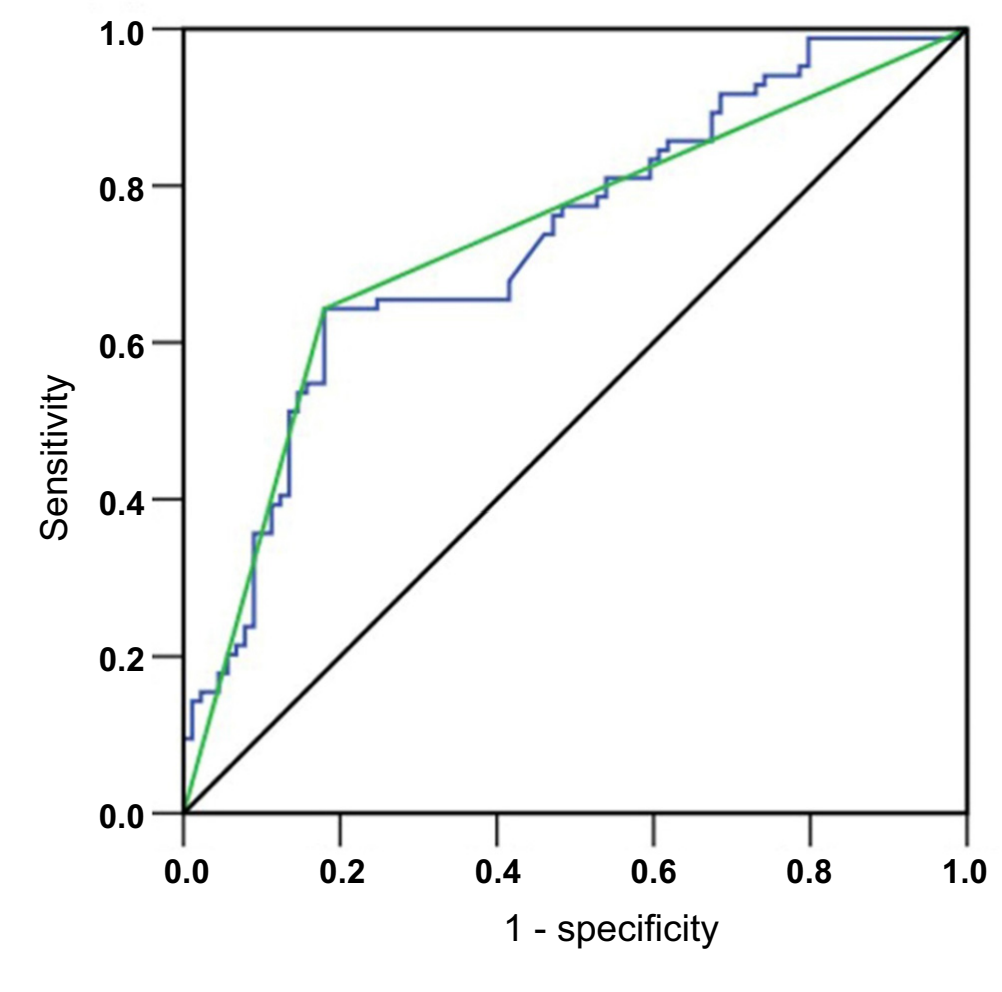
**Prediction value of the SMS in the verification group according to AP severity.** The blue line represents a continuous variable and the green line represents categorical variable. The AUCs were 0.741 and 0.739, respectively. AP: Acute pancreatitis; AUC: Area under the curve; SMS: Stroke mobility score.

We included data from patients from FMUUH as an external validation dataset to verify the predictive ability of SMS. In the FMUUH population, there were 106 non-severe cases and 67 severe cases. Non-severe patients had fewer SIRS, fewer cases of organ dysfunction within 48 h, and fewer ICU admissions than severe patients. There were no significant differences in other demographic and pathological characteristics, such as age, sex, American Society of Anesthesiologists (ASA) score, and BMI (*P* > 0.05, Table S3). The AUCs of AP severity in the validation group were 0.741 and 0.739 in the form of SMS continuous variables and categorical variables, respectively, showing stable predictive efficacy (Figure 3).

## Discussion

This multicenter study investigated the relationship between changes in body composition during treatment and the degree of AP severity. The results showed that PreSMR and ΔSMI were predictors of AP severity. In this study, we established a new assessment tool, the SMS, based on these two indicators, which can accurately predict AP severity. The measurement of muscle indices during AP treatment is expected to be a simple and effective method of evaluating therapeutic effects and assessing patient prognosis.

Most patients with AP find relief after standard treatment; however, a subset may progress to SAP, thus experiencing serious local or systemic complications, which leads to high mortality and sequelae. Therefore, it is important to identify patients predisposed to SAP early. Early, aggressive monitoring and nutritional support in these patients can reduce the incidence of complications and curb pancreatitis-related mortality [16]. In predicting the severity of AP, previous studies have often relied on the comprehensive evaluation of clinical signs, hematological indicators, and the degree of organ dysfunction [17]. However, these indicators are limited by low specificity and delayed prediction. In contrast, it has been shown that significant changes in patients’ body compositions during treatment are associated with the risk of death and both local and systemic complications of AP [18]. It has also been reported that the BMI or waist circumference may be associated with AP progression [19]; however, combining the BMI with other predictive models (such as APACHE-II or Apache-obesity [Apache-O]) does not significantly improve the predictive efficacy [20]. Consequently, some researchers argue that this parameter has a limited role in predicting AP severity. The results of this study support this view as there were no significant differences in BMI between patients with non-SAP and those with SAP. This suggests that BMI measurements, which cannot differentiate between fat and muscle tissue, may be less relevant. Instead, the distribution of fat and muscle may be a more important determinant of patient prognosis than the amount of fat [21]. Nevertheless, sarcopenia has been associated with poor prognosis, complications, and increased pancreatitis incidence [10]. Therefore, we utilized indicators of dynamic changes in body composition during treatment, such as ΔSMI, to dynamically assess AP severity.

Current studies indicate that inflammatory cells have strong metabolic requirements because of their rapid proliferation. The ability to adjust metabolic activity to meet energy and biosynthesis needs throughout disease progression is crucial for the survival of these cells. During disease progression, these cells undergo significant metabolic rewiring to adapt to their high energy demands and changing environmental conditions [22]. Recent research has confirmed that changes in patients’ metabolic indices (including BMI, low-density lipoprotein, and total cholesterol) are associated with the prognoses of patients with acute cholecystitis [23]. Assessing body composition parameters, such as fat and muscle tissue, can be used to assess a patient’s energy reserves and expenditure.

In 2012, the Acute Pancreatitis Classification Working Group modified the Atlanta Classification System to enhance the evaluation and treatment of AP. The revised Atlanta classification emphasizes AP morphology with radiology playing an increasingly vital role in the evaluation and follow-up of patients with this condition [24]. CT is the primary method for assessing and monitoring AP and is also the most widely used technique for evaluating body composition, considered the gold standard for measuring fat and muscle tissue [25].

Utilizing CT, we analyzed and measured body composition parameters, finding that PreSMR and ΔSMI are significant predictors of AP severity. The ΔSMI in the non-severe group was notably higher than that in the severe group, whilst the SMR in the non-severe group was lower than in the severe group. A decrease in skeletal muscle mass may increase the likelihood of progression from AP to SAP and elevate mortality risk. This aligns with previous studies investigating the relationship between skeletal muscle depletion and severe illnesses, including an examination of the impact of skeletal muscle depletion on ICU patients. For instance, Weijs et al. [26] identified a low skeletal muscle area as a mortality risk factor, irrespective of ICU admission indication. Kortebein et al. [27] demonstrated that just ten days of bed rest could significantly reduce skeletal muscle mass.

The pathophysiological process of AP often involves metabolic disorders. Yang et al. [28] observed that patients with SAP exhibit an increased release of inflammatory factors and a significant decrease in amino acid levels such as leucine and arginine. This limited the tricarboxylic acid cycle, urea cycle, and transamination metabolism, thereby leading to protein synthesis and energy metabolism deficiencies. Lower initial skeletal muscle content can exacerbate muscle mass depletion under the physiological stress of metabolic disorders associated with AP. This may be linked to leptin, known for its role in modulating inflammation [29, 30]. Muscle wasting or decreased skeletal muscle function can disrupt leptin’s inflammatory modulation, increasing AP risk. This is supported by studies showing that exogenous leptin can attenuate the inflammatory response in AP models [31].

Consequently, we developed a new assessment tool, the SMS, by combining ΔSMI and SMR, which significantly enhances the accuracy of predicting AP severity, with an AUC of 0.764. In the external verification group, the AUC of 0.741 was achieved, demonstrating the model’s applicability to different populations and its strong universality. Therefore, clinicians should measure ΔSMI and PreSMR using CT during treatment to provide timely, individualized care to patients.

Nevertheless, this study had some limitations. First, as this was a retrospective study, selection bias was inevitable. However, prospective data collection and external validation cohorts should have reduced this bias. Second, detailed information on the nutritional support of patients during AP treatment was not available in this study, which may have affected the results. Therefore, further studies are required to improve this model.

## Conclusion

AP causes changes in body composition, and ΔSMI and PreSMR during AP treatment can accurately predict the severity of the condition. It is recommended to routinely evaluate patients with AP using the predictive model obtained in this study for individualized diagnosis and treatment.

## Supplemental data

**Table S1:** Changes in body components before and after treatment

**Variables**	**Median [IQR]**	**Pearson correlation**	***P* value**
Body mass index, kg/m^2^		0.781	0.331
Before treatment	25.9 [23.5–28.2]		
After treatment	25.5 [23.5–27.9]		
Difference	−0.4 [−0.9–1.2]		
Subcutaneous adipose tissue, cm^2^/m^2^		0.892	0.539
Before treatment	29.5 [15.1–44.7]		
After treatment	28.2 [18.3–43.2]		
Difference	−1.3 [−6.3–3.3]		
Visceral adipose tissue, cm^2^/m^2^		0.880	0.919
Before treatment	21.3 [5.9–41.2]		
After treatment	20.3 [9.9–39.1]		
Difference	−1.0 [−6.4–6.5]		
Skeletal muscle index, cm^2^/m^2^		0.878	0.507
Before treatment	47.2 [40.7–51.9]		
After treatment	46.7 [41.2–53.2]		
Difference	−0.5 [−3.2–2.3]		
Skeletal muscle radiodensity, HU		0.615	0.363
Before treatment	44.0 [40.3–49.5]		
After treatment	44.6 [39.7–48.7]		
Difference	0.6 [−3.6–4.6]		

IQR: Interquartile range.

**Table S2:** Multifactor logistic regression analysis for AP severity prediction

**Variables**	**Univariate analysis**		**Multivariate analysis**	
	**Hazard ratio (95% CI)**	***P* value**	**Hazard ratio (95% CI)**	***P* value**
Age	1.034 (0.998–1.071)	0.061		
Sex		0.311		
Male	Reference			
Female	0.659 (0.295–1.475)			
ASA score		0.226		
≤2	Reference			
>2	1.514 (0.774–2.962)			
Body mass index		0.917		
≤25 kg/m^2^	Reference			
>25 kg/m^2^	1.077 (0.267–4.350)			
Diabetes	1.429 (0.538–3.796)	0.594		
Hypertension	1.690 (0.743–3.844)	0.362		
Smoking	0.909 (0.174–4.746)	0.462		
WBC	2.975 (1.404–4.683)	0.040	1.203 (0.347–0.165)	0.163
HCT	2.286 (0.710–7.362)	0.128		
CRP	0.635 (0.327–1.236)	0.181		
PCT	0.235 (0.112–2.103)	0.412		
Blood amylase	0.827 (0.627–2.821)	0.329		
Blood lipase	0.615 (0.411–1.120)	0.211		
Blood calcium	1.236 (0.569–5.778)	0.685		
ΔSMI		0.016		0.032
≥1.0 cm^2^/m^2^	Reference		Reference	
<1.0 cm^2^/m^2^	0.418 (0.206–0.851)		0.534 (0.283–0.834)	
PreSMR		<0.001		0.005
≥43.7 HU	Reference		Reference	
<43.7 HU	0.402 (0.198–0.882)		0.643 (0.322–0.890)	

ASA: American Society of Anesthesiologists; WBC: White blood cells; HCT: Hematocrit; CRP: C-reactive protein; PCT: Procalcitonin; ΔSMI: The difference between the SMI before and after treatment; PreSMR: The SMR before treatment; SMI: Skeletal muscle index; SMR: Skeletal muscle radiodensity; AP: Acute pancreatitis; Reference: a group with baseline characteristics that was used as a comparison.

**Table S3:** Demographic and pathological characteristics of the external validation dataset

**Variables**	**Non-severe group (*n* ═ 106)**	**Severe group (*n* ═ 67)**	***P* value**
Age, mean (SD), years	57 (8.5)	55 (8.6)	0.189
Sex, *n* (%)			0.358
Male	66 (62.3%)	37 (55.2%)	
Female	40 (37.7%)	30 (44.8%)	
ASA score, *n* (%)			0.064
≤2	79 (74.5%)	41 (61.2%)	
>2	27 (25.5%)	26 (38.8%)	
Body mass index, *n* (%)			0.336
≤25 kg/m^2^	49 (46.2%)	36 (53.7%)	
>25 kg/m^2^	57 (53.8%)	31 (46.3%)	
Diabetes, *n* (%)	39 (36.8%)	21 (31.3%)	0.463
Hypertension, *n* (%)	41 (38.7%)	27 (40.3%)	0.832
Smoking, *n* (%)	38 (35.8%)	27 (40.3%)	0.556
WBC, mean (SD), ×10^9^/L	13.42 (5.49)	13.88 (5.21)	0.585
HCT, mean (SD), %	42.01 (7.81)	40.92 (7.16)	0.357
CRP, mean (SD), mg/L	79.30 (51.84)	76.74 (49.06)	0.747
PCT, mean (SD), ng/mL	3.74 (3.27)	4.33 (3.75)	0.063
Blood amylase, mean (SD), U/L	288.4 (182.3)	297.7 (176.1)	0.741
Blood lipase, mean (SD), U/L	362.5 (67.9)	366.0 (86.6)	0.767
Blood calcium, mean (SD), mmol/L	2.07 (0.20)	1.97 (0.31)	0.101
Etology, *n* (%)			0.585
Alcohol	37 (34.9%)	17 (25.4%)	
Biliary	38 (35.8%)	30 (44.8%)	
Hypertriglyceridemia	3 (2.8%)	1 (1.5%)	
Drug-induced	3 (2.8%)	1 (1.5%)	
Idiopathic	25 (23.6%)	18 (26.9%)	
Severity outcome			
Persistent SIRS after 48h, *n* (%)	25 (23.6%)	26 (38.8%)	0.032
Persistent organ failure after 48 h, *n* (%)	18 (17.0%)	23 (34.3%)	0.009
Need for ICU admission, *n* (%)	22 (20.8%)	28 (41.8%)	0.003
Length of stay, median [IQR], days	15 [5–25]	14 [5–29]	0.792
In-hospital mortality, *n* (%)	5 (4.7%)	4 (6.0%)	0.718
30 days unplanned readmission, *n* (%)	13 (12.3%)	15 (22.4%)	0.078
Interventions performed, *n* (%)			0.194
Percutaneous puncture drainage	31 (29.2%)	21 (31.3%)	
Endoscopic transluminal drainage/necrosectomy	4 (3.8%)	7 (10.4%)	
Open necrosectomy	2 (1.9%)	2 (3.0%)	

ASA: American Society of Anesthesiologists; WBC: White blood cells; HCT: Hematocrit; CRP: C-reactive protein; PCT: Procalcitonin.
